# Structural Heart: The Journal of the Heart Team - Starting a New Era

**DOI:** 10.1016/j.shj.2025.100722

**Published:** 2025-10-13

**Authors:** Josep Rodés-Cabau

**Affiliations:** a Quebec Heart & Lung Institute, Laval University, Quebec City, Quebec, Canada; b Department of Research and Innovation, Clínic Barcelona, Barcelona, Spain

It is with great enthusiasm that I begin my role as Editor-in-Chief of *Structural Heart: The Journal of the Heart Team*, the official journal of Cardiovascular Research Foundation (CRF). Together with a renewed team of associate and deputy editors, I embrace this responsibility with a deep sense of commitment and a clear vision to further the journal’s growth and global impact. Our goal is to strengthen *Structural Heart’s* position as one of the most influential and respected journals in the field of structural cardiovascular interventions.

First and foremost, I extend my sincere gratitude to Dr. Anthony DeMaria for his exceptional leadership over the past 8 years. Under his guidance, *Structural Heart* evolved into a well-established, widely recognized journal with thousands of monthly manuscript views and a remarkable 2025 Impact Factor of 2.8 (Q2). We inherit a strong foundation upon which we are ready to build. While we will maintain many of the journal’s core features and sections, several updates will be introduced to reflect the vision of our new editorial team and CRF’s broader mission.

## Reinforcing Our Educational Mission

Education remains central to *Structural Heart* and CRF. We aim to publish a greater number of high-quality review articles and expert consensus statements, with a strong emphasis on mentoring the next generation of researchers. We encourage young investigators to collaborate with experienced thought leaders to contribute to these works.

## Launching the “Innovation Corner”

The pace of innovation in structural heart interventions is unprecedented. To support and highlight this progress, we are introducing a new section: the Innovation Corner. This section will include preclinical, first-in-human, and early feasibility studies, as well as studies on significant device iterations and new indications for existing technologies. Importantly, this section will also be linked with innovation sessions at CRF meetings, offering authors the opportunity to simultaneously present and publish their findings.

## Introducing “Trial Design in the Structural Heart Field”

With the growing number of pivotal trials reshaping clinical practice in the structural heart field, understanding trial design is more important than ever. The design of such trials remains a major aspect for better understanding their targeted population as well as the potential trial strengths and limitations. This is key when applying the results of randomized trials in real-world clinical practice. Thus, a new section, Trial Design in the Structural Heart Field, will be added to the journal, and we encourage the investigators of pivotal trials to submit this type of manuscript to *Structural Heart* in the future.

## Expanding the Scope Beyond Valvular Interventions

While valve therapies remain central, the field of structural heart interventions encompasses much more: from interventional therapies for thromboembolic prevention (e.g., left atrial appendage occlusion, patent foramen ovale closure) to therapies for chronic and acute heart failure, systemic and pulmonary hypertension, and congenital heart diseases. We aim for *Structural Heart* to reflect this full spectrum and invite submissions across all noncoronary cardiovascular interventions.

## Enhancing Dissemination Through TCTMD

Reaching a global audience is crucial. Disseminating the results of published studies and reaching the maximum number of potential readers remains of utmost importance. CRF is well recognized as one of the most influential organizations worldwide regarding knowledge dissemination and education in the interventional field, particularly through several yearly meetings and the TCTMD platform. I am pleased to announce a new partnership with TCTMD, which will spotlight key original articles from each issue through features and author interviews. These efforts will amplify the visibility and reach of your work, with more dissemination initiatives in development—stay tuned.

## Improving Speed and Efficiency

We are committed to accelerating the editorial process. Our goal is a submission-to-decision time of under 30 days for all submissions, and we are streamlining the path from acceptance to PubMed Central indexing. If you have recently submitted a manuscript, you may already be seeing some of these improvements, with more to come.

## Embracing a Global Perspective

Currently, the vast majority of journal submissions come from the United States. As we grow, we aim to increase the contributions from all regions worldwide. The composition of our new Editorial Board reflects this international outlook, and we invite researchers from around the world to consider *Structural Heart* as their journal of choice.

In parallel with the exponential growth of structural heart interventions, a new era has begun for *Structural Heart*. You—the authors, reviewers, and readers—are at the heart of this transformation. With your partnership and the continued strong support of the CRF, we look forward to an exciting journey ahead. We are ready to work tirelessly to support your work, share your studies, and drive the future of structural heart disease treatment. Let us shape the next chapter together.

## Funding

The author has no funding to report.

## Disclosure Statement

The author reports no conflict of interest regarding the content of this article.

